# Correction: Thiazole-fused androstenone and ethisterone derivatives: potent β- and γ-actin cytoskeleton inhibitors to treat melanoma tumors

**DOI:** 10.1039/d5md90014j

**Published:** 2025-04-03

**Authors:** Sanjay Adhikary, Subrata Roy, Shailesh Budhathoki, Siam Chowdhury, Abbey Stillwell, Alexei G. Basnakian, Alan Tackett, Nathan Avaritt, Mohamed Milad, Mohammad Abrar Alam

**Affiliations:** a Department of Chemistry and Physics, College of Sciences and Mathematics, Arkansas State University Jonesboro Arkansas 72467 USA malam@astate.edu; b Environmental Sciences Program, College of Sciences and Mathematics, Arkansas State University Jonesboro AR 72467 USA; c Molecular Biosciences Program, College of Sciences and Mathematics, Arkansas State University Jonesboro AR 72467 USA; d Computer Science, The College of Engineering and Computer Science, Arkansas State University Jonesboro AR 72468 USA; e Department of Pharmacology and Toxicology, University of Arkansas for Medical Sciences 4301 W. Markham St Little Rock AR 72205 USA; f Central Arkansas Veterans Healthcare System W. 7th St Little Rock AR 72205 USA; g Department of Biochemistry and Molecular Biology, University of Arkansas for Medical Sciences Little Rock AR 72205 USA; h The Department of Mathematics and Statistics, Arkansas State University Jonesboro AR 72467 USA; i Arkansas Biosciences Institute, Arkansas State University Jonesboro AR 72467 USA

## Abstract

Correction for ‘Thiazole-fused androstenone and ethisterone derivatives: potent β- and γ-actin cytoskeleton inhibitors to treat melanoma tumors’ by Sanjay Adhikary *et al.*, *RSC Med. Chem.*, 2025, **16**, 1105–1130, https://doi.org/10.1039/d4md00719k.

The authors regret that the name displayed for compound **E17** in the ‘Materials and methods’ section of their article contains an error. The corrected name for compound **E17** is (1*S*,2*R*,13*R*,14*S*,17*R*,18*S*)-17-ethynyl-2,18-dimethyl-7-[(3-methyl-2-pyridyl)amino]-8-thia-6-azapentacyclo[11.7.0.0^2,10^.0^5,9^.0^14,18^]icosa-5(9),6,10-trien-17-ol.

The Royal Society of Chemistry apologises for these errors and any consequent inconvenience to authors and readers.

